# A rapid *in vitro* assay for evaluating the effects of acetylcholinesterase inhibitors and reactivators in the rat basolateral amygdala

**DOI:** 10.3389/fncel.2022.1066312

**Published:** 2022-11-21

**Authors:** Jeffrey S. Thinschmidt, Scott W. Harden, Michael A. King, James D. Talton, Charles J. Frazier

**Affiliations:** ^1^Department of Pharmacodynamics, College of Pharmacy, University of Florida, Gainesville, FL, United States; ^2^Alchem Laboratories Corporation, Alachua, FL, United States

**Keywords:** organophosphates, basolateral amygdala, acetylcholinesterase, status epilepticus, NIMP, HI-6, electrophysiology, acetylcholine

## Abstract

We established a novel brain slice assay to test the ability of acetylcholinesterase (AChE) reactivators to prevent ACh-induced M1 muscarinic acetylcholine receptor (mAChR) dependent hyperexcitability observed after exposure to the organophosphate (OP)-based AChE inhibitor and sarin surrogate 4-nitrophenyl isopropyl methylphosphonate (NIMP). Whole-cell patch clamp recordings were used to evaluate the response of pyramidal neurons in the rat basolateral amygdala (BLA) to brief (1 min) bath application of ACh (100 μM), either in control conditions, or after exposure to NIMP ± an AChE reactivator. Bath application of ACh produced atropine- and pirenzepine-sensitive inward currents in voltage clamped BLA pyramidal neurons, and increased the frequency of spontaneous EPSCs, suggesting robust activation of M1 mAChRs. Responses to ACh were increased ~3–5 fold in slices that had been preincubated in NIMP, and these effects were reversed in a concentration dependent manner by exposure to a commercially available AChE reactivator. The current work outlines a simple assay that can be used to evaluate the efficacy of both known and novel AChE reactivators in an area of the limbic system that likely contributes to seizures after acute exposure to OP-based AChE inhibitors.

## Introduction

Organophosphates (OPs) were widely used as an active agents in pesticides in the mid-20th century and continue to be used in chemical weapons. These compounds phosphorylate the serine in the active site of acetylcholinesterase (AChE) preventing it from being able to hydrolyze acetylcholine (ACh, Meek et al., 2012; Mercey et al., 2012). In people with acute exposure to toxic levels of OP-based nerve agents and pesticides, inhibition of AChE produces dangerous effects in both the peripheral and central nervous system (Albuquerque et al., 2006; Abou-Donia et al., 2016). In the peripheral nervous system, buildup of ACh in the neuromuscular junction promotes inactivation of postsynaptic nicotinic acetylcholine receptors, disrupting voluntary movement, promoting apnea, and ultimately causing respiratory failure. In the central nervous system, OP-based toxins promote loss of consciousness and seizures, and can cause lasting brain damage secondary to both cholinergic and glutamatergic excitotoxicity. These effects of acute exposure to OP-based nerve agents are often lethal. Prior to exposure, one preventative countermeasure involves controlled delivery of a reversible (non-OP based) AChE inhibitor, such as pyridostigmine bromide. After acute OP-poisoning, the best available therapeutics to counteract peripheral effects are cholinesterase reactivators (and oximes) such as 2-pyridine aldoxime methyl chloride (2-PAM), and asoxime chloride (HI-6). Unfortunately, these agents have low permeability to the blood brain barrier (BBB), resulting in poor efficacy against central effects of OP-poisoning, which can remain debilitating and potentially lethal. A number of alternative strategies to address toxic effects of OP based nerve agents in the CNS have been explored. Often they involve muscarinic acetylcholine receptor antagonists (Carpentier et al., 2000; Miller et al., 2015), allosteric modulators of GABA_A_ receptors (McDonough et al., 1999; Reddy, 2016), and/or specific types of glutamate receptor antagonists (Figueiredo et al., 2011; Myhrer et al., 2013; Miller et al., 2015; Aroniadou-Anderjaska et al., 2019; Lumley et al., 2021). While there remains potential for forward progress in these areas, overall, there is still a clear need for discovery and/or development of novel AChE reactivators that are both permeant to the BBB and effective in the CNS.

The goal of the current study was to develop an *in vitro* assay capable of reliably quantifying the effects of OPs and AChE reactivators in the brain. We chose the basolateral amygdala (BLA) as the central location for these studies because it receives dense cholinergic innervation from the basal forebrain (Unal et al., 2015), is rich in expression of AChE (Woolf and Butcher, 1982; Kellis et al., 2020), and is often a focal point for seizures in patients with temporal lobe epilepsy (Aroniadou-Anderjaska et al., 2008). Further, the BLA is known to contribute directly to status epilepticus as produced by exposure to the OP-based AChE inhibitor soman, through a mechanism that involves inhibition of AChE (Apland et al., 2009; Prager et al., 2013). Our results reveal excitatory effects of ACh on individual BLA pyramidal neurons, and on local excitatory transmission, that are dependent on activation of M1 mAChRs and are strongly enhanced by an OP-based AChE inhibitor. We further demonstrate reversal of AChE inhibition with a commercially available reactivator. Overall, this study describes a novel approach to quantifying the effects of AChE inhibitors and reactivators in the CNS, and in so doing strongly reinforces what appears to be a prominent role of the BLA in producing central effects of acute OP-poisoning. We expect the experimental approach described here may be of significant utility in evaluating the effects of novel AChE reactivators in the future.

## Materials and Methods

### Animals

Male Sprague Dawley rats (p16–p30, Envigo, Indianapolis, IN) were used for all experiments. Standard rodent chow (Teklad 7912, Envigo) and water was available *ad libitum*. Housing was 20°C–26°C and 30%–70% relative humidity. All procedures were approved by the Institutional Animal Care and Use Committee at the University of Florida.

### Acute brain slice preparation

Animals were anesthetized with an IP injection of ketamine/xylazine (100/10 mg/kg). Following a lack of paw pinch withdraw reflex, animals were rapidly decapitated. The brain was removed, and submerged in an ice-cold sucrose-laden dissecting solution that contained (in mM): 205 sucrose, 10 D-glucose, 1 MgSO_4_, 2 KCl, 1.25 NaH_2_PO_4_, 1 CaCl_2_, and 25 NaHCO_3_ saturated with 95% O_2_/5% CO_2_. Brains were then blocked to isolate the region containing the BLA and 300 μm thick coronal sections were made using a Leica VT 1200s vibratome. Slices were then hemisected and transferred to an incubation chamber filled with an ACSF containing (in mM): 124 NaCl, 10 D-glucose, 3 MgSO_4_, 2.5 KCl, 1.23 NaH_2_PO_4_, 1 CaCl_2_, and 25 NaHCO_3_, which was saturated with 95% O_2_/5% CO_2_, and maintained at 35°C. After 30 min, slices were allowed to passively equilibrate to room temperature (for minimum of 30 min) prior to use.

### Preexposure to AChE inhibitors and reactivators

Incubation with physostigmine, NIMP, and HI-6 was performed using a custom 6-well tissue culture plate (Fisher Scientific part #07-200-83), with the ACSF for slice incubation (described above) as a vehicle. Well volumes were 5–10 ml and wells were outfitted with polyethylene tubing to deliver carbogen (95% O_2_, 5% CO_2_) to 2–3 brain slices per well. Unless otherwise noted, slices were incubated ≥2 h with NIMP or physostigmine and were then transferred to a recording chamber containing standard ACSF (without NIMP). In cases where slices were exposed to an AChE reactivator, this occurred immediately after exposure to NIMP, for at least 30 min prior to whole cell recording. After all preincubation protocols were complete, slices were transferred to a recording chamber and all whole-cell recordings were performed in standard ACSF that did not contain any AChE inhibitors or reactivators. Cholinergic agonists and antagonists were applied acutely, in the recording chamber, via the perfusion system. ACh, atropine, pirenzepine, and physostigmine were purchased from Tocris (Minneapolis, MN). NIMP was purchased from Accela Chembio Inc. (San Diego, CA). HI-6 was purchased from Sigma-Aldrich (St. Louis, MO).

### Whole-cell patch clamp recording and analysis

Experiments were performed in standard ACSF that contained (in mM): 126 NaCl, 11 D-glucose, 1.5 MgSO_4_, 3 KCl, 1.2 NaH_2_PO_4_, 2.4 CaCl_2_, and 25 NaHCO_3_. This solution was saturated with 95% O_2_/5% CO_2_, was maintained at 30°C using an inline heater (Warner Instruments, TC-324B), and was continuously delivered to the recording chamber at 2 ml/min. Patch pipettes were pulled using a Sutter Instruments Flaming/Brown P-97 puller, and had an open tip resistance of 4–6 MΩ when filled with an internal solution containing (in mM): 115 K-gluconate, 10 phosphocreatine, 10 HEPES, 0.5 EGTA, 2 MgCl_2_, 4 Na_2_ ATP, 0.4 Na_3_ GTP, and 5 KCl. This solution was passed through a 0.22 μm filter prior to use. Osmolarity was adjusted to 295 mOsm, and pH was adjusted to 7.3.

Slices were imaged using an Olympus BX51 WI upright microscope using infrared differential interference contrast (DIC) microscopy. Data were acquired at 20 kHz using a Multiclamp 700 B amplifier, a Digidata 1440 A digitizer, and Clampex software version 10.2 (Molecular Devices). Pyramidal cells were identified for whole-cell recording by their location, shape, and size as apparent in the DIC image, and by their intrinsic electrical properties once recording was initiated. During recording, holding current, membrane resistance, access resistance, and whole-cell capacitance were monitored at 0.16 Hz in voltage clamped neurons using responses to brief voltage steps from −70 to −80 mV. Neurons were discarded from analysis if access resistance changed by >30 MΩ during the course of experiments. In control conditions BLA pyramidal neurons used in this study had mean whole-cell capacitance of 124.7 ± 10.84 pF, and mean membrane resistance of 183.40 MΩ. Small and/or fast firing interneurons were excluded from the study. Holding current and sESPC frequency reported in all figures were measured in BLA pyramidal neurons voltage clamped at −70 mV. Baseline values were obtained in the 2 min prior to acute (1 min) bath application of ACh. ACh-induced changes in holding current and sEPSC frequency reported are the mean of values observed during a 1 min period immediately after ACh application. In rare cases where it was necessary to capture the maximal effect (e.g., see Figure 5), ACh-induced changes reported are mean values observed in the second minute after ACh application. Spontaneous events were identified and event parameters were quantified using parameter-based event detection software written in OriginC by CJF (OriginLab, Northampton, MA). Holding current was measured independently of spontaneous synaptic events using techniques previously described (e.g., see Nahir et al., 2007; Harden and Frazier, 2016; Pati et al., 2020).

**Figure 1:**
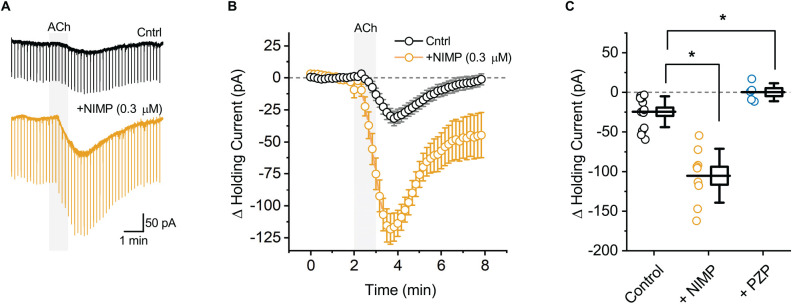
ACh-induced changes in holding current in BLA pyramidal neurons can be used to quantify the effects of central AChE inhibition. **(A)** Representative holding current traces from BLA pyramidal neurons illustrating the response to acute bath application of 100 μM ACh (shaded region) in control conditions (black) and following exposure to NIMP (0.3 μM, orange). Rapid transients observed in these traces were produced by a membrane test delivered every 10 s (see “Materials and methods” Section). **(B)** ACh-induced change in holding current over time as recorded in neurons from control slices (*n* = 14, open black circles) and in neurons from slices preincubated in 0.3 μM NIMP (open orange circles, *n* = 9). ACh was bath applied from 2 to 3 min (gray shaded area). Error bars represent the SEM. **(C)** Summary data illustrating the ACh-induced change in holding current, observed from 3 to 4 min as plotted in panel **(B)**, for each cell in the control and NIMP datasets (open black and orange circles respectively). Blue circles illustrate the effect of ACh on holding current in neurons from control slices, as observed in the presence of 1 μM PZP. For each group, box height illustrates the SEM, whiskers illustrate the SD, and horizontal lines highlight the group means. Asterisks indicate *p* < 0.05.

**Figure 2:**
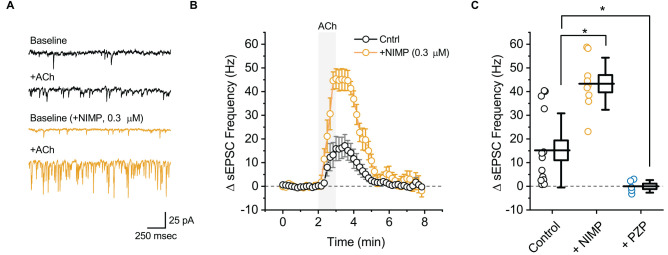
ACh-induced increases in sEPSC frequency in BLA pyramidal neurons can be used to quantify the effects of central AChE inhibition. **(A)** Top two traces (black) illustrate the effect of bath applied ACh (100 μM for 1 min) on sEPSCs as observed in a representative BLA pyramidal neuron voltage clamped at −70 mV in control conditions. Next two traces (orange) are representative of data obtained when an identical experiment was performed in a slice that was preincubated in 0.3 μM NIMP. **(B)** ACh-induced change in sEPSC frequency over time as recorded in neurons from control slices (*n* = 14, open black circles) and from neurons in slices preincubated in 0.3 μM NIMP (open orange circles, *n* = 9). ACh (100 μM) was bath applied from 2 to 3 min (gray shaded area). Error bars represent the SEM. **(C)** Summary data illustrating the ACh-induced change in sEPSC frequency, observed from 3 to 4 min as plotted in panel **(B)**, for each cell in the control and NIMP datasets (open black and orange circles respectively). Blue circles (PZP) illustrate the effect of ACh on sEPSC frequency in neurons from control slices, as observed in the presence of 1 μM PZP. For each group, box height illustrates the SEM, whiskers illustrate the SD, and horizontal lines highlight the group means. Asterisks indicate *p* < 0.01.

**Figure 3:**
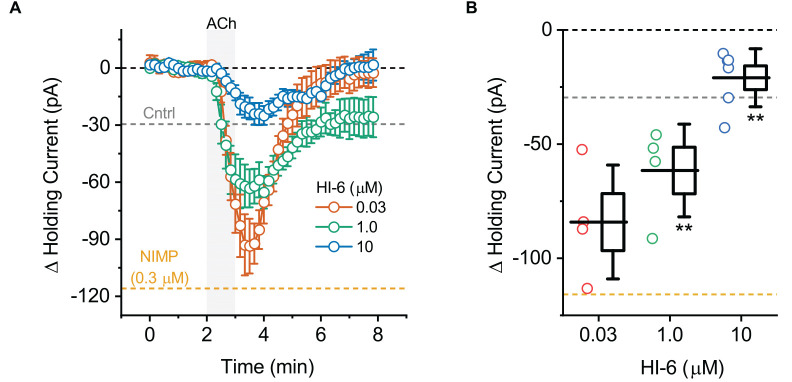
ACh-induced changes in holding current in BLA pyramidal neurons can be used to quantify the effectiveness of AChE reactivators in slices preincubated with NIMP. **(A)** ACh-induced change in holding current over time as recorded in BLA pyramidal neurons from slices preincubated in 0.3 μM NIMP, and then treated with various concentrations of AChE reactivator HI-6 as indicated in the legend. **(B)** Summary data illustrating the ACh-induced change in holding current, observed from 3 to 4 min as plotted in panel **(A)**, for each cell tested. Group colors are matched to the panel **(A)** legend. For each group, box height illustrates the SEM, whiskers illustrate the SD, and horizontal lines highlight the group means. In both panels, the dashed gray and orange lines highlight the mean ACh-induced change in holding current as observed at 3–4 min in BLA pyramidal neurons from control and NIMP datasets as illustrated in Figure 1B. Dashed black line highlights 0 pA. **Indicates *p* ≤ 0.05 compared to responses observed in slices treated identically with NIMP but not exposed to HI-6. See text of “Results” Section and Table 1 for further details.

**Table 1:** Summary of ACh-induced changes in holding current, sEPSC frequency, and sEPSC amplitude across multiple experiments.

Condition	N	Δ iHold (pA)	Δ sEPSC Freq (Hz)	Δ sEPSC Amp (pA)
**Control**	14	−24.5, 5.2	15.2 ± 4.2	0.49 ± 0.6
**Pirenzepine (1 μM)**	5	0.2 ± 5.1*	−0.03 ± 1.2**	−1.6 ± 1.2
**Atropine (25 μM)**	4/6	2.4 ± 4.5*	0.2 ± 1.7**	0.4 ± 1.0
**NIMP (μM)**				
0.01	6	−20.6 ± 7.3	9.6 ± 5.9	0.1 ± 0.7
0.3	9	−105.3 ± 11.3**	43.3 ± 3.7**	3.2 ± 1.2*
10	8	−120.9 ± 31.8*	45.0 ± 3.6**	7.6 ± 1.2**
**Physostigmine (100 μM)**	4	−61.2 ± 12.0**	33.7 ± 1.2**	6.9 ± 4.7
**HI-6 (μM)**				
0.03	4	−84.1 ± 12.5	41.3 ± 9.7	8.1 ± 4.5
1	4	−61.6 ± 10.2^†^	28.8 ± 7.6	2.4 ± 2.4
10	6	−20.9 ± 5.2^††^	8.9 ± 3.6^††^	−0.1 ± 0.7

**Figure 4:**
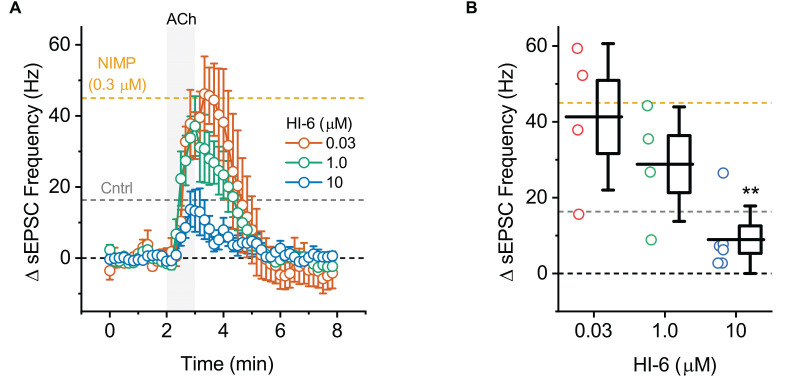
ACh-induced increases in sEPSC frequency in BLA pyramidal neurons can be used to quantify the effectiveness of AChE reactivators in slices preincubated with NIMP. **(A)** ACh-induced change in sEPSC frequency over time as produced by acute bath application of ACh in BLA pyramidal neurons preincubated in 0.3 μM NIMP, and then treated with various concentrations of AChE reactivator HI-6 as indicated in the legend. See “Results”/”Materials and methods” Section for further details. **(B)** Summary data illustrating the ACh-induced change in sEPSC frequency, observed from 3 to 4 min as plotted in panel **(A)**, for each cell tested. For each group, box height illustrates the SEM, whiskers illustrate the SD, and horizontal lines highlight the group means. In both panels, the dashed gray and orange lines highlight the mean ACh-induced change in sEPSC frequency as observed at 3–4 min in BLA pyramidal neurons from control and NIMP datasets as illustrated in Figure 2B. Dashed black line highlights 0 pA. **Indicates *p* ≤ 0.05 compared to responses observed in slices treated identically with NIMP but not exposed to HI-6. See text of results and Table 1 for further details.

*p < 0.05, **p < 0.01 (vs. Control); ^†^*p* < 0.05, ^†^^†^*p* < 0.01 (vs. 0.3 μM NIMP). NIMP, physostigmine, and HI-6 were all applied to slices during incubation period (prior to whole-cell recording) as described in the “Materials and methods” Section. Values are the peak (mean ± SEM) measured during minutes 3–4 as illustrated in Figures 1–4.

**Figure 5:**
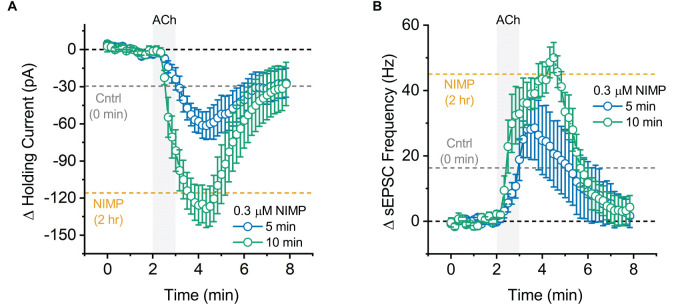
Maximal effect of 0.3 μM NIMP on ACh-evoked responses is apparent after 10 min, but not 5 min, of preincubation. **(A)** ACh-induced change in holding current over time as produced by acute bath application of ACh in BLA pyramidal neurons preincubated in 0.3 μM NIMP for 5 vs. 10 min (open blue vs. open green circles respectively). **(B)** ACh-induced change in sEPSC frequency produced by acute bath application of ACh in BLA pyramidal neurons preincubated in 0.3 μM NIMP for 5 vs. 10 min (open blue vs. open green circles respectively). In both panels, the dashed gray and orange lines highlight the mean ACh-induced response as observed at 3–4 min in BLA pyramidal neurons from control and 0.3 μM NIMP datasets as illustrated in Figures 1; 2B. Dashed black lines highlight 0 pA or 0 Hz. Error bars represent the SEM.

### Statistics

Standard paired and unpaired *t*-tests were used to compare two population means as observed within or across groups, respectively. For comparisons that involved >2 population means, a standard one-way ANOVA was used, with Holm-Sidak *post-hoc* tests when appropriate.

## Results

### The AChE inhibitor NIMP enhances M1-mediated excitation of BLA pyramidal neurons following acute bath application of ACh

BLA pyramidal neurons were targeted for whole-cell recording in acute tissue slices using their unique morphological and electrophysiological features (see “Materials and methods” Section). Voltage-clamp experiments were performed at −70 mV to monitor changes in holding current induced by acute (1 min) bath application of ACh. We found that ACh reliably produced a downward shift in holding current, indicative of excitation (Figures 1A,B, black traces, ΔiHold: −24.5 ± 5.2 pA, *t* = 4.69, *p* = 4.2 × 10^−4^, *n* = 14). This effect was blocked by pretreatment with the non-selective mAChR antagonist atropine (25 μM, ΔiHold: 2.4 ± 4.5 pA, *t* = 0.54, *p* = 0.63, *n* = 4). It was also blocked by pretreatment with the selective M1 receptor antagonist pirenzepine (PZP, 1 μM, Figure 1C, ΔiHold: 0.2 ± 5.1 pA, *t* = 0.04, *p* = 0.97, *n* = 5). These data indicate that acute bath application of ACh induces an M1 receptor dependent decrease in holding current in BLA pyramidal neurons.

ACh-induced changes in holding current were then evaluated in acute brain slices that had been preincubated for 2 h in 0.3 μM NIMP (see “Materials and methods” Section). We found that prior exposure to 0.3 μM NIMP significantly increased the ACh-induced change in holding current (Figures 1A,B, orange traces, ΔiHold: −105.3 ± 11.3 pA, *n* = 9, *t* = 7.27, *p* = 3.67 × 10^−7^ vs. control slices not exposed to NIMP).

Preincubation of naive slices in either 10 μM or 0.01 μM (instated of 0.3 μM) NIMP demonstrated that the effects of NIMP were concentration dependent. A one-way ANOVA used to compare the mean ACh-evoked change in holding current observed across all NIMP concentrations tested (0, 0.01, 0.3, and 10 μM) revealed a clear effect of NIMP concentration (*F* = 11.30, *p* = 2.96 × 10^−5^). *Post-hoc* tests highlighted that the ACh-induced change in holding current was significantly enhanced compared to controls (0 μM NIMP) in slices preincubated with both 0.3 and 10 μM NIMP, but not in slices preincubated in 0.01 μM NIMP (*t* = 4.04, 4.64, 0.17, *p* = 3.03 × 10^−4^, 5.25 × 10^−5^, 0.87, respectively). *Post-hoc* tests also revealed there was no significant difference in the ACh-evoked response as observed in slices pretreated with 0.3 vs. 10 μM NIMP (*t* = 0.69, *p* = 0.50). Collectively, these data indicate that bath applied ACh acts on M1 mAChRs to produce robust excitatory effects in BLA pyramidal neurons, and further indicates that prior exposure to the OP-based AChE inhibitor NIMP significantly enhances this effect of ACh in a concentration dependent manner, with maximal effects observed at a concentration of ≥ 0.3 μM (for further details also see Table 1).

### The AChE inhibitor NIMP enhances M1-mediated increases in sEPSC frequency observed in BLA pyramidal neurons following acute bath application of ACh

In order to evaluate the effect of ACh on local excitatory signaling within the BLA, we used parameter-based event detection software to quantify ACh-induced changes in the frequency of spontaneous excitatory postsynaptic currents (sEPSC), as observed in the same recordings that were used to quantify the ACh-induced change in holding current described above. In control conditions, acute bath application of ACh increased sEPSC frequency in BLA pyramidal neurons by 15.2 ± 4.2 Hz (Figures 2A,B, black traces, *t* = 3.62, *p* = 3.12 × 10^−3^, *n* = 14). Similar to the effects on holding current, the effect of ACh on sEPSC frequency was blocked by atropine (ΔsEPSC freq: 0.2 ± 1.7 Hz, *t* = 0.15, *p* = 0.89, *n* = 6), was blocked by PZP (ΔsEPSC freq: −0.03 ± 1.2 Hz, *n* = 5, *t* = 0.02, *p* = 0.98), and was substantially enhanced in slices that had been preincubated in 0.3 μM NIMP (Figures 2A,B, orange traces, ΔsEPSC freq: 43.3 ± 3.7 Hz, *n* = 9, *t* = 4.68, *p* = 1.27 × 10^−4^ vs. control).

A one-way ANOVA was used to compare the mean ACh-evoked change in sEPSC frequency observed across all NIMP concentrations tested (0, 0.01, 0.3, and 10 μM). This analysis indicated a main effect of NIMP concentration (*F* = 16.29, *p* = 1.14 × 10^−6^), while *post-hoc* tests revealed that the ACh-induced change in sEPSC frequency was significantly enhanced compared to controls in slices preincubated with both 0.3 and 10 μM NIMP, but not in slices preincubated in 0.01 μM NIMP (*t* = 4.93, 5.03, 0.85, *p* = 2.29 × 10^−5^, 1.69 × 10^−6^, 0.40, respectively). Additional *post-hoc* tests revealed there were no significant differences in the ACh-evoked increase in sEPSC frequency as observed in slices pretreated with 0.3 vs. 10 μM NIMP (*t* = 0.26, *p* = 0.80). Collectively, these data demonstrate that acute bath application of ACh produces a clear increase in sEPSC frequency in BLA pyramidal neurons. They further highlight that this response depends on activation of M1 mAChRs, and is significantly enhanced in slices preincubated in NIMP (with maximal effects observed at a concentration of ≥ 0.3 μM, see Table 1 for further details).

It is worth highlighting that in each case, results obtained when analyzing ACh-induced changes in sEPSC frequency mirror those obtained when analyzing ACh-induced changes in holding current. Interestingly, we further noted that ACh had no significant effect on sEPSC amplitude in control conditions (+0.49 ± 0.6 pA, *t* = 0.78, *p* = 0.44), and yet produced a small but statistically significant increase in slices exposed to both 0.3 and 10 μM NIMP (+3.2 ± 1.2 pA, *n* = 9, *t* = 2.77 *p* = 0.02, +7.75 ± 1.24 pA, *t* = 6.22, *p* = 4.35 × 10^−4^, respectively, see Table 1 for additional details). We expect this is likely due to recruitment of a small number of previously silent perisomatic synapses during acute application of ACh in slices treated with ≥ 0.3 μM NIMP.

Finally, in order to determine whether a non-OP based AChE inhibitor would produce similar effects in our assay, we tested 100 μM physostigmine (2-h preincubation) using identical techniques and found that it also significantly enhanced ACh-evoked changes in holding current and sEPSC frequency relative to controls (ΔiHold: −61.15 ± 12.01 pA, *n* = 4, *t* = 3.17, *p* = 6.00 × 10^−3^, ΔsEPSC freq: 33.66 ± 1.16 Hz, *n* = 4, *t* = 4.25, *p* = 7.25 × 10^−4^, Table 1). However, ACh-induced responses observed in slice preincubated in 100 μM physostigmine were significantly smaller than those observed in slices preincubated in 0.3 μM NIMP (for ΔiHold: *t* = 2.32, *p* = 0.04, for ΔsEPSC freq: *t* = 2.51, *p* = 0.03, *n* = 4, 9 in both cases, see Table 1 for further details).

### Treatment with the AChE reactivator HI-6 reverses the effects of NIMP on M1 mediated responses following bath application of ACh in BLA pyramidal neurons

To evaluate the ability of this assay to quantify the effectiveness of AChE reactivators, experiments demonstrating that preincubation with NIMP enhances subsequent ACh-evoked changes in holding current (Figure 1) and sEPSC frequency (Figure 2) were repeated in brain slices preincubated for 2 h in 0.3 μM NIMP and then further exposed for 30 min to the AChE reactivator HI-6, at a concentration of 0, 0.03, 1.0, or 10 μM.

A one-way ANOVA that evaluated the ACh-induced change in holding current in these experiments indicated a clear effect of HI-6 concentration (*F* = 12.81, *p* = 8.26 × 10^−5^, Figures 3A,B). *Post-hoc* tests further revealed that ACh-evoked responses as observed in slices pretreated with 0.3 μM NIMP were significantly reduced in slices that were further treated with either 10 μM or 1.0 μM HI-6, but not in slices treated with 0.03 μM HI-6 (*t* = 6.08, 2.76, 1.34, *p* = 7.60 × 10^−6^, 0.01, 0.20, respectively). An additional *post-hoc* test revealed that the ACh-induced change in holding current observed in slices exposed to 10 μM HI-6 was significantly smaller than the response observed in slices exposed to 1 μM HI-6 (*t* = 2.39, *p* = 0.03, Table 1), indicating that the maximal effect of HI-6 requires a concentration >1.0 μM.

A one-way ANOVA that evaluated the ACh-induced change in sEPSC frequency in these same experiments also indicated a clear effect of HI-6 concentration (*F* = 9.47, *p* = 4.88 × 10^−4^, Figures 4A,B). However, in this case, *post-hoc* tests indicated a significant reduction in the ACh-evoked response only in slices that had been exposed to 10 μM HI-6 (after 0.3 μM NIMP, *t* = 5.06, *p* = 6.99 × 10^−5^). By contrast, the ACh-evoked response in slices exposed to 0.3 or 1.0 μM HI-6, after 0.3 μM NIMP, did not significantly differ from slices exposed to NIMP alone (*t* = 1.87, 0.26, *p* = 0.08, 0.80, respectively).

Collectively these results reveal that a 30 min exposure to 10 μM HI-6 can effectively reduce the impact of 0.3 μM NIMP on ACh-evoked responses in BLA pyramidal neurons, as reported by changes in both holding current and sEPSC frequency. Indeed, when directly compared, ACh-induced responses observed in slices exposed to both NIMP and 10 μM HI-6 were not significantly different than those observed in control slices that were not treated with NIMP or HI-6 (ΔiHold: *t* = 0.48, *p* = 0.69, ΔsEPSC freq: *t* = 1.13, *p* = 0.028, also see Table 1). These results also demonstrate that HI-6 is an effective AChE reactivator in BLA slices previously exposed to an OP-based AChE inhibitor, and highlight that the assay developed here is an efficient and effective way to quantify the effect of AChE reactivators on both cellular and synaptic function in the BLA.

### Maximal effects of NIMP are observed after approximately 10 min of exposure

In a final series of experiments, we evaluated the time required for preincubation in 0.3 μM NIMP to effectively enhance ACh-evoked responses. A one-way ANOVA evaluating ACh-evoked changes in holding current observed across all NIMP preincubation times used (0 min, 5 min, 10 min, and 2 h) revealed a main effect of incubation time (*F* = 20.04, *p* = 1.41 × 10^−7^). *Post-hoc* tests indicated NIMP preincubation produced a significant increase in the ACh-evoked response at all times tested (relative to no NIMP exposure, *t* = 2.31, 6.58, 6.08, *p* = 0.03, 1.75 × 10^−7^, 7.60 × 10^−7^, for 5 min, 10 min, and 2 h, respectively). One additional *post-hoc* test highlighted that there was no significant difference between effects observed with 10 min vs. 2-h preincubation (*t* = 0.89, *p* = 0.38). See Figure 5A and legend for further details.

Similarly, a one-way ANOVA evaluating ACh-induced increases in sEPSC frequency across these same groups also indicated a main effect of incubation time (*F* = 9.73, *p* = 9.53 × 10^−5^). *Post-hoc* tests again indicated that the maximal effect of NIMP was apparent with just 10 min preincubation (10 min vs. 0 min: *t* = 4.26, *p* = 1.59 × 10^−4^, 2 h vs. 0 min: *t* = 4.56, *p* = 6.78 × 10^−5^, 10 min vs. 2 h, *t* = 0.05, *p* = 0.96). Interestingly, however, in this case 5 min of preincubation was not sufficient to significantly enhance the ACh-evoked response (*t* = 1.58, *p* = 0.12). See Figure 5B and legend for further details. Collectively, these results highlight that relatively brief exposure to 0.3 μM NIMP is sufficient to produce near maximal effect on ACh-evoked responses as observed in BLA pyramidal neurons.

## Discussion

We describe a novel, rapid, and reproducible assay useful for efficiently quantifying the effects of AChE inhibitors and reactivators *in vitro*, using acute brain slices through the BLA. During the development of this assay, we chose to use nitrophenyl isopropyl methylphosphonate (NIMP) as the AChE inhibitor, the oxime HI-6 as a reactivator, and the basolateral amygdala as the CNS location of interest. NIMP was chosen as the AChE inhibitor because it is a commercially available OP-based sarin surrogate that phosphorylates AChE at the same site as sarin, and because prior work has indicated its effectiveness in CNS preparations (Ohta et al., 2006; Meek et al., 2012; Chambers et al., 2016; Dail et al., 2019; Chambers and Meek, 2020; Angrand et al., 2021). HI-6 was chosen as the AChE reactivator because it is a commercially available oxime with known efficacy against peripheral effects of OP-poisoning (Antonijevic and Stojiljkovic, 2007; Myhrer et al., 2018). The BLA was chosen as the CNS site of interest because it receives dense cholinergic innervation, is rich in expression of AChE, is often a focal point for seizures in patients with temporal lobe epilepsy, and because it has been directly implicated in seizures as produced by OP-poisoning (Woolf and Butcher, 1982; Aroniadou-Anderjaska et al., 2008, 2009; Prager et al., 2013; Unal et al., 2015).

Notably, the majority of prior work directly implicating the BLA in the central effects of acute OP-poisoning has relied heavily on *in vivo* exposure to OPs and extracellular recoding of neural activity, combined with molecular assays of AChE activity and/or neuronal damage, at various timepoints post exposure (in addition to above see also Apland et al., 2010, 2017; Figueiredo et al., 2011; Prager et al., 2014; Miller et al., 2015). It is worth highlighting that a significant subset of the work noted above has compared results observed in BLA to those in hippocampus, and broadly concluded that although the hippocampus is also highly susceptible to OP-poisoning, OPs produce significantly more neuronal damage in BLA, and development of status epilepticus *in vivo* requires significant reduction of AChE activity in the BLA (e.g., see Aroniadou-Anderjaska et al., 2009; Prager et al., 2013). Broadly speaking, the techniques used in the studies cited above were quite appropriate for the primary goals; namely for identifying key sites of OP action in the CNS, and for revealing their relationship to OP-induced seizures as observed *in vivo*. That said, with the importance of the BLA in mediating central effects of OPs established, it is now desirable to have a method for generating repeatable, reliable, measures of the effects of exogenous AChE inhibitors and reactivators on both neuronal and network physiology in the BLA. Methods employed in prior studies were less well optimized for that goal, and thus that is the primary need we have attempted to address in the current study.

For development of the assay presented here, we used acute bath application of exogenous ACh as a stimulus, in both control and NIMP treated slices, and we chose to measure responses using whole-cell patch clamp recordings from individual voltage clamped BLA pyramidal neurons, rather than with sharp electrode or extracellular field recordings. Our use of a reliable, repeatable, and well-controlled cholinergic stimulus represents a significant difference from prior studies in this area. It is an important difference because it allows us to measure the effect of AChE inhibitors in the absence of any dependence on the basal level of endogenous cholinergic neurotransmission in the slice. This is important because the basal level of endogenous acetylcholine release can reasonably be expected to vary substantially between slices, and is likely to be quite low in preparations where axons of cholinergic efferents have been severed (which includes typical preparations of both BLA and hippocampus). We expect this type of issue has contributed to the high variability and long latency typically noted in prior studies that have attempted to directly measure physiological responses to acute application of AChE inhibitors *in vitro* (often in hippocampus, e.g., see Endres et al., 1989; Harrison et al., 2004, 2005; Kozhemyakin et al., 2010; Spencer et al., 2010; Alkondon et al., 2013). Similarly, our choice to measure whole-cell responses to acute application of ACh in voltage clamped BLA pyramidal neurons maximized our ability to simultaneously and yet independently measure direct cholinergic excitation of BLA pyramidal neurons, and related increases in local glutamatergic signaling likely to directly participate in acute and chronic ictogenesis. These effects were reported by ACh-induced changes in holding current and sEPSC frequency respectively.

The data presented here indicate that inhibition of AChE with the OP-based AChE inhibitor NIMP produced an approximate 5-fold increase in the ability of ACh application to produce M1 receptor mediated changes in holding current, and a near 3-fold increase in the ability of ACh to produce M1 receptor mediated increases in sEPSC frequency. Qualitatively similar effects were produced with the non-OP based AChE inhibitor physostigmine, confirming these effects are likely due to direct inhibition of AChE. Importantly, the current study also demonstrates that NIMP induced inhibition of AChE was successfully reversed by treatment with known AChE reactivator HI-6, and that reactivation can be measured reliably by monitoring the effects of ACh on holding current and sEPSC frequency in BLA pyramidal neurons. Further experiments revealed maximal effects of NIMP on BLA pyramidal neurons can be produced by exposure to concentrations as low as 0.3 μM for as little as 10 min. Notably, the effects of brief exposure to NIMP were not observed to wash-out at any time during the experimental day. This result is consistent with the expectation that, in the absence of the effect of an AChE reactivator, the effects OP-based AChE inhibitors are irreversible. That idea, in turn, reinforces the conclusion that the effects of HI-6 reported here were not due to the additional time required to apply it.

More broadly, the results of this study confirm conclusions of prior studies in highlighting the extreme sensitivity of the BLA to OP-poisoning, and in demonstrating the effectiveness of HI-6 as a reactivator of OP-inhibited AChE in the CNS. However, as noted in the introduction, HI-6 has poor permeability to the blood brain barrier, and thus limited ability in a clinical setting to reverse dangerous central effects of OP-poisoning. In that regard, one limitation of the assay developed here is that it does not provide a means to evaluate BBB permeability of new putative AChE reactivators. Another potential limitation is that in focusing on cellular and network level effects, the current assay does not directly evaluate potential genomic effects of AChEIs on cholinergic signaling systems, which may be relevant to better understanding not only of acute OP poisoning, but also the effects of lower doses of AChEIs delivered as therapeutics for various types of neurocognitive disorders (e.g., see Kaufer et al., 1998, 1999; Dulawa and Janowsky, 2019). That said, in our view the method presented here should have immediate and significant value as an efficient preclinical tool for reliably and rapidly quantifying the ability of novel AChE reactivators to restore the effects of endogenous AChE in the BLA, hippocampus, and elsewhere in the CNS. Novel reactivators that are highly effective in this assay should then be further evaluated for both acute and long-term effects in more intact systems.

## Data Availability Statement

The original contributions presented in the study are included in the article, further inquiries can be directed to the corresponding author.

## Ethics Statement

The animal study was reviewed and approved by Institutional Animal Care and Use Committee at the University of Florida, and was further confirmed to be compliant with Federal and Department of Defense guidelines by the Animal Care and Use Office of the USARMDC.

## Author Contributions

JST developed the assay, performed experiments, completed data analysis, and wrote the initial version of the manuscript. SWH assisted with data analysis and manuscript editing. MAK assisted with manuscript editing. JDT assisted with direction of the project and manuscript editing. CJF assisted with direction of the project, assay development, manuscript writing, production of final figures, and development of software tools for data analysis. All authors contributed to the article and approved the submitted version.

## Funding

This work was sponsored by the U.S. Government under Other Transaction number W15QKN-16-9-1002 between the MCDC, and the Government. The US Government is authorized to reproduce and distribute reprints for Governmental purposes notwithstanding any copyright notation thereon.

## Conflict of Interest

JDT was employed by the company Alchem Laboratories Corporation. MAK was a consultant for the company Alchem Laboratories Corporation. The remaining authors declare that the research was conducted in the absence of any commercial or financial relationships that could be construed as a potential conflict of interest.

## Publisher’s Note

All claims expressed in this article are solely those of the authors and do not necessarily represent those of their affiliated organizations, or those of the publisher, the editors and the reviewers. Any product that may be evaluated in this article, or claim that may be made by its manufacturer, is not guaranteed or endorsed by the publisher.

## References

[B1] Abou-Donia M. B. Siracuse B. Gupta N. Sokol A. S. (2016). Sarin (GB, O-isopropyl methylphosphonofluoridate). neurotoxicity: critical review. Crit. Rev. Toxicol. 46, 1–31. 10.1080/10408444.2016.1220916 27705071 PMC5764759

[B2] Albuquerque E. X. Pereira E. F. R. Aracava Y. Fawcett W. P. Oliveira M. Randall W. R.. (2006). Effective countermeasure against poisoning by organophosphorus insecticides and nerve agents. Proc. Nat. Acad. Sci. U S A 103, 13220–13225. 10.1073/pnas.0605370103 16914529 PMC1550772

[B3] Alkondon M. Albuquerque E. X. Pereira E. F. R. (2013). Acetylcholinesterase inhibition reveals endogenous nicotinic modulation of glutamate inputs to CA1 stratum radiatum interneurons in hippocampal slices. Neurotoxicology 36, 72–81. 10.1016/j.neuro.2013.02.005 23511125 PMC5673493

[B4] Angrand L. Takillah S. Malissin I. Berriche A. Cervera C. Bel R.. (2021). Persistent brainwave disruption and cognitive impairment induced by acute sarin surrogate sub-lethal dose exposure. Toxicology 456:152787. 10.1016/j.tox.2021.152787 33887375

[B5] Antonijevic B. Stojiljkovic M. P. (2007). Unequal efficacy of pyridinium oximes in acute organophosphate poisoning. Clin. Med. Res. 5, 71–82. 10.3121/cmr.2007.701 17456837 PMC1855336

[B6] Apland J. P. Aroniadou-Anderjaska V. Braga M. F. M. (2009). Soman induces ictogenesis in the amygdala and interictal activity in the hippocampus that are blocked by a GluR5 kainate receptor antagonist *in vitro*. Neuroscience 159, 380–389. 10.1016/j.neuroscience.2008.11.053 19136046 PMC2947795

[B7] Apland J. P. Aroniadou-Anderjaska V. Figueiredo T. H. Prager E. M. Olsen C. H. Braga M. F. M. (2017). Susceptibility to soman toxicity and efficacy of LY293558 against soman-induced seizures and neuropathology in 10-month-old male rats. Neurotox. Res. 32, 694–706. 10.1007/s12640-017-9789-7 28776308

[B8] Apland J. P. Figueiredo T. H. Qashu F. Aroniadou-Anderjaska V. Souza A. P. Braga M. F. M. (2010). Higher susceptibility of the ventral versus the dorsal hippocampus and the posteroventral versus anterodorsal amygdala to soman-induced neuropathology. Neurotoxicology 31, 485–492. 10.1016/j.neuro.2010.05.014 20570628 PMC2933957

[B9] Aroniadou-Anderjaska V. Figueiredo T. H. Apland J. P. Braga M. F. (2019). Targeting the glutamatergic system to counteract organophosphate poisoning: a novel therapeutic strategy. Neurobiol. Dis. 133:104406. 10.1016/j.nbd.2019.02.017 30798006

[B10] Aroniadou-Anderjaska V. Figueiredo T. H. Apland J. P. Qashu F. Braga M. F. M. (2009). Primary brain targets of nerve agents: the role of the amygdala in comparison to the hippocampus. Neurotoxicology 30, 772–776. 10.1016/j.neuro.2009.06.011 19591865 PMC2761531

[B11] Aroniadou-Anderjaska V. Fritsch B. Qashu F. Braga M. F. M. (2008). Pathology and pathophysiology of the amygdala in epileptogenesis and epilepsy. Epilepsy Res. 78, 102–116. 10.1016/j.eplepsyres.2007.11.011 18226499 PMC2272535

[B12] Carpentier P. Foquin A. Rondouin G. Lerner-Natoli M. de Groot D. M. Lallement G. (2000). Effects of atropine sulphate on seizure activity and brain damage produced by soman in guinea-pigs: ECoG correlates of neuropathology. Neurotoxicology 21, 521–540. 11022861

[B13] Chambers J. E. Meek E. C. (2020). Central neuroprotection demonstrated by novel oxime countermeasures to nerve agent surrogates. Ann. N Y Acad. Sci. 1479, 5–12. 10.1111/nyas.14352 32319115 PMC9513985

[B14] Chambers J. E. Meek E. C. Chambers H. W. (2016). Novel brain-penetrating oximes for reactivation of cholinesterase inhibited by sarin and VX surrogates. Ann. N Y Acad. Sci. 1374, 52–58. 10.1111/nyas.13053 27153507 PMC4940271

[B15] Dail M. B. Leach C. A. Meek E. C. Olivier A. K. Pringle R. B. Green C. E.. (2019). Novel brain-penetrating oxime acetylcholinesterase reactivators attenuate organophosphate-induced neuropathology in the rat hippocampus. Toxicol. Sci. 169, 465–474. 10.1093/toxsci/kfz060 30835286 PMC6542341

[B16] Dulawa S. C. Janowsky D. S. (2019). Cholinergic regulation of mood: from basic and clinical studies to emerging therapeutics. Mol. Psychiatry 24, 694–709. 10.1038/s41380-018-0219-x 30120418 PMC7192315

[B17] Endres W. Spuler A. Bruggencate G. t. (1989). Acetylcholinesterase reactivators antagonize epileptiform bursting induced by paraoxon in guinea pig hippocampal slices. J. Pharmacol. Exp. Ther. 251, 1181–1186. 2600810

[B18] Figueiredo T. H. Qashu F. Apland J. P. Aroniadou-Anderjaska V. Souza A. P. Braga M. F. M. (2011). The GluK1 (GluR5). Kainate/α-Amino-3-hydroxy-5-methyl-4-isoxazolepropionic acid receptor antagonist LY293558 reduces soman-induced seizures and neuropathology. J. Pharmacol. Exp. Ther. 336, 303–312. 10.1124/jpet.110.171835 20962029 PMC3033714

[B19] Harden S. W. Frazier C. J. (2016). Oxytocin depolarizes fast-spiking hilar interneurons and induces GABA release onto mossy cells of the rat dentate gyrus. Hippocampus 26, 1124–1139. 10.1002/hipo.22595 27068005 PMC5237430

[B20] Harrison P. K. Sheridan R. D. Green A. C. Scott I. R. Tattersall J. E. H. (2004). A guinea pig hippocampal slice model of organophosphate-induced seizure activity. J. Pharmacol. Exp. Ther. 310, 678–686. 10.1124/jpet.104.065433 15031302

[B21] Harrison P. K. Sheridan R. D. Green A. C. Tattersall J. E. H. (2005). Effects of anticonvulsants on soman-induced epileptiform activity in the guinea-pig *in vitro* hippocampus. Eur. J. Pharmacol. 518, 123–132. 10.1016/j.ejphar.2005.06.032 16054127

[B22] Kaufer D. Friedman A. Seidman S. Soreq H. (1998). Acute stress facilitates long-lasting changes in cholinergic gene expression. Nature 393, 373–377. 10.1038/30741 9620801

[B23] Kaufer D. Friedman A. Seidman S. Soreq H. (1999). Anticholinesterases induce multigenic transcriptional feedback response suppressing cholinergic neurotransmission. Chem. Biol. Interact. 119, 349–360. 10.1016/s0009-2797(99)00046-0 10421471

[B24] Kellis D. M. Kaigler K. F. Witherspoon E. Fadel J. R. Wilson M. A. (2020). Cholinergic neurotransmission in the basolateral amygdala during cued fear extinction. Neurobiol. Stress 13:100279. 10.1016/j.ynstr.2020.100279 33344731 PMC7739185

[B25] Kozhemyakin M. Rajasekaran K. Kapur J. (2010). Central cholinesterase inhibition enhances glutamatergic synaptic transmission. J. Neurophysiol. 103, 1748–1757. 10.1152/jn.00949.2009 20107127 PMC2853275

[B26] Lumley L. A. Marrero-Rosado B. Rossetti F. Schultz C. R. Stone M. F. Niquet J.. (2021). Combination of antiseizure medications phenobarbital, ketamine and midazolam reduces soman-induced epileptogenesis and brain pathology in rats. Epilepsia Open 6, 757–769. 10.1002/epi4.12552 34657398 PMC8633481

[B370] McDonough J. H. Zoeffel L. D. McMonagle J. Copeland T. L. Smith C. D. Shih T.-M. (1999). Anticonvulsant treatment of nerve agent seizures: anticholinergics versus diazepam in soman-intoxicated guinea pigs. Epilepsy Res. 38, 1–14. 10.1016/S0920-1211(99)00060-1 10604601

[B27] Meek E. C. Chambers H. W. Coban A. Funck K. E. Pringle R. B. Ross M. K.. (2012). Synthesis and *in vitro* and *in vivo* inhibition potencies of highly relevant nerve agent surrogates. Toxicol. Sci. 126, 525–533. 10.1093/toxsci/kfs013 22247004

[B29] Mercey G. Verdelet T. Renou J. Kliachyna M. Baati R. Nachon F.. (2012). Reactivators of acetylcholinesterase inhibited by organophosphorus nerve agents. Acc. Chem. Res. 45, 756–766. 10.1021/ar2002864 22360473

[B30] Miller S. L. Aroniadou-Anderjaska V. Figueiredo T. H. Prager E. M. Almeida-Suhett C. P. Apland J. P.. (2015). A rat model of nerve agent exposure applicable to the pediatric population: the anticonvulsant efficacies of atropine and GluK1 antagonists. Toxicol. Appl. Pharm. 284, 204–216. 10.1016/j.taap.2015.02.008 25689173 PMC4545593

[B31] Myhrer T. Mariussen E. Aas P. (2018). Development of neuropathology following soman poisoning and medical countermeasures. Neurotoxicology 65, 144–165. 10.1016/j.neuro.2018.02.009 29454886

[B32] Myhrer T. Mariussen E. Enger S. Aas P. (2013). Capacities of metabotropic glutamate modulators in counteracting soman-induced seizures in rats. Eur. J. Pharmacol. 718, 253–260. 10.1016/j.ejphar.2013.08.024 24021536

[B33] Nahir B. Bhatia C. Frazier C. J. (2007). Presynaptic inhibition of excitatory afferents to hilar mossy cells. J. Neurophysiol. 97, 4036–4047. 10.1152/jn.00069.2007 17442771

[B34] Ohta H. Ohmori T. Suzuki S. Ikegaya H. Sakurada K. Takatori T. (2006). New safe method for preparation of sarin-exposed human erythrocytes acetylcholinesterase using non-toxic and stable sarin analogue isopropyl p-nitrophenyl methylphosphonate and its application to evaluation of nerve agent antidotes. Pharmaceut. Res. 23, 2827–2833. 10.1007/s11095-006-9123-1 17096183

[B35] Pati D. Harden S. W. Sheng W. Kelly K. B. Kloet AD d. e. Krause E. G.. (2020). Endogenous oxytocin inhibits hypothalamic CRH neurons following acute hypernatremia. J. Neuroendocrinol. 32:e12839. 10.1111/jne.12839 32133707 PMC7384450

[B36] Prager E. M. Aroniadou-Anderjaska V. Almeida-Suhett C. P. Figueiredo T. H. Apland J. P. Braga M. F. M. (2013). Acetylcholinesterase inhibition in the basolateral amygdala plays a key role in the induction of status epilepticus after soman exposure. Neurotoxicology 38, 84–90. 10.1016/j.neuro.2013.06.006 23817175

[B37] Prager E. M. Aroniadou-Anderjaska V. Almeida-Suhett C. P. Figueiredo T. H. Apland J. P. Rossetti F.. (2014). The recovery of acetylcholinesterase activity and the progression of neuropathological and pathophysiological alterations in the rat basolateral amygdala after soman-induced status epilepticus: relation to anxiety-like behavior. Neuropharmacology 81, 64–74. 10.1016/j.neuropharm.2014.01.035 24486384 PMC4005290

[B1600] Reddy D. S. (2016). Neurosteroids for the potential protection of humans against organophosphate toxicity. Ann. N Y Acad. Sci. 1378, 25–32. 10.1111/nyas.13160 27450921 PMC5063687

[B38] Spencer J. P. Middleton L. J. Davies C. H. (2010). Investigation into the efficacy of the acetylcholinesterase inhibitor, donepezil and novel procognitive agents to induce gamma oscillations in rat hippocampal slices. Neuropharmacology 59, 437–443. 10.1016/j.neuropharm.2010.06.005 20600173

[B39] Unal C. T. Pare D. Zaborszky L. (2015). Impact of basal forebrain cholinergic inputs on basolateral amygdala neurons. J. Neurosci. 35, 853–863. 10.1523/JNEUROSCI.2706-14.2015 25589777 PMC4293427

[B40] Woolf N. J. Butcher L. L. (1982). Cholinergic projections to the basolateral amygdala: a combined Evans Blue and acetylcholinesterase analysis. Brain Res. Bull. 8, 751–763. 10.1016/0361-9230(82)90102-2 6182963

